# α-Mangostin Exhibits Antitumor Activity Against NCI-H1975 Cells via the EGFR/STAT3 Pathway: An Experimental and Molecular Simulation Study

**DOI:** 10.3390/molecules30061294

**Published:** 2025-03-13

**Authors:** Jing Wang, Jiamin Xian, Ruohan Zhang, Zhuoyi Wang, Shuanggou Zhang, Die Zhao, Jun Sheng, Peiyuan Sun

**Affiliations:** 1 Key Laboratory of Pu-er Tea Science, Ministry of Education, Yunnan Agricultural University, Kunming 650201, China; 18214585880@163.com (J.W.); xjm9798@163.com (J.X.); 18160229352@163.com (Z.W.); zhang15012106135@163.com (S.Z.); 2 College of Science, Yunnan Agricultural University, Kunming 650201, China; mariposai@163.com; 3 College of Food Science and Technology, Yunnan Agricultural University, Kunming 650201, China; 15215003760@163.com

**Keywords:** NSCLC, EGFR, α-Mangostin, antitumor activity, molecular simulation

## Abstract

Non-small cell lung cancer (NSCLC) harboring epidermal growth factor receptor (EGFR) mutations have brought great challenges to the medical treatment in the world. Current treatment strategies, such as EGFR tyrosine kinase inhibitors (TKIs), have reached certain achievements, however, patients inevitably experienced resistance after undergoing a period of treatment with these drugs. Hence, more novel therapy strategies need to be urgently developed. Natural compounds have become popular topics in drug development. α-Mangostin, which is derived from mangosteen, possesses multiple biological properties, yet the antitumor mechanism against NSCLC has not been further elucidated. In this study, an MTT assay, Western blotting, a colony formation assay, and flow cytometry were performed to detect the antitumor activity of α-Mangostin on NSCLC cell NCI-H1975. Molecular docking and molecular dynamics simulations were performed to analyze the interactions between α-Mangostin and the core target proteins. The results indicated that α-Mangostin exerts its antitumor activity by inhibiting cell proliferation and migration, reducing cell cycle arrest, promoting cell apoptosis, and regulating the phosphorylation expression levels of EGFR and signal transducer and activator of transcription 3 (STAT3). Moreover, the results of the molecular simulation study revealed the potential binding mode of α-Mangostin to EGFR and STAT3. In summary, we characterized that α-Mangostin may be used as a potent pro-drug against NSCLC via the EGFR/STAT3 pathway.

## 1. Introduction

Lung cancer, which is divided into non-small cell lung cancer (NSCLC, accounting for almost 85% of confirmed cases) and small cell lung cancer (SCLC, accounting for almost 15% of confirmed cases), is one of the deadliest diseases in the world. In 2022, according to incomplete statistics from the American Cancer Society, there were 236,740 new cases and 130,180 deaths in both sexes [1,2,3]. As the classic target against NSCLC, epidermal growth factor receptor (EGFR) is a member of the ErbB (HER) family, EGFR binds to ligands through its extracellular domain, inducing conformational changes that activate the receptor. The intracellular domain contains a tyrosine kinase domain, which catalyzes the phosphorylation of tyrosine residues on substrates, thereby activating downstream signaling pathways [4], and its abnormal activation plays an important role in physiological processes of NSCLC, including proliferation, differentiation, invasion, cell cycle and resistance to apoptosis [5]. Furthermore, the EGFR activating mutation has brought great challenges to the treatment against NSCLC. Despite the advent of EGFR tyrosine kinase inhibitors (TKIs), which successfully treat NSCLC harboring EGFR mutations, most patients inevitably developed resistance to these agents after a period of treatment [6]. There is an urgent need for the development of more novel therapeutic strategies against NSCLC. Signal transducer and activator of transcription 3 (STAT3), as one of the key proteins in EGFR downstream pathways, is persistently activated in over 50% of patients with NSCLC, and its increased expression is associated with poor tumor differentiation, advanced clinical stage, lymph node metastasis, and drug resistance [7,8,9,10,11,12]. Mutations in receptor tyrosine kinases such as EGFR have been implicated in constitutively activated STAT3 signaling in NSCLC. Receptor tyrosine kinases such as EGFR have intrinsic kinase domains and directly phosphorylate STAT3 following the binding to ligands [13]. Hence, inhibition of EGFR and STAT3 are critical in the treatment against NSCLC.

Natural products extracted from vegetables, fruits, and herbal medicines have been the main focus of the research because of their low toxicity, side effects, resistance, and low metastasis rate [14]. In this study, α-Mangostin was found to exhibit inhibitory activity against NSCLC cell NCI-H1975. The compound is isolated from the pericarps of mangosteen, which is known as the “Queen of Fruits” and is cultivated in Yunnan, Guangdong and Hainan Province of China [15]. α-Mangostin is the amplest xanthone in the pericarps of mangosteen and possesses multiple pharmacological properties, such as antitumor activity and antioxidant activity [16,17,18,19,20,21,22,23]. Additionally, it is reported that the agent exerts protective effects against cisplatin-induced renal injury [24,25]. Its molecular structure contains a typical xanthone core, with multiple substituents such as alkyl and hydroxyl groups attached. The presence and position distribution of these substituents influence the physicochemical properties and biological activities of α-Mangostin, endowing it with certain lipophilicity and a specific spatial configuration, enabling it to interact with various targets in organisms [26]. α-Mangostin is usually a yellow or light yellow crystalline powder, soluble in organic solvents such as methanol, ethanol, acetone, and chloroform, but has relatively low solubility in water [27]. This solubility characteristic gives it certain advantages in the extraction and separation processes using organic solvents. Meanwhile, it provides a reference for its application in pharmaceutical preparations. A large number of studies have found that α-Mangostin can inhibit the mutation of mutant isocitrate dehydrogenase 1 (IDH1). Somatic heterozygous mutations of this protein have been detected in various cancers and can regulate cancer cell metabolism [28]. α-Mangostin is also proven to possess anti-lung cancer activity, yet its mechanism warrants further investigation [29].

With the development of computational biology, to further assess the molecular mechanism by which active components ameliorate diseases, more and more researchers have employed computational approaches, such as molecular docking and molecular dynamics simulation [30]. Among them, as an important method in computer-aided drug design, molecular docking predicts the molecular recognition mechanism between a ligand and a receptor by simulating the binding mode of their three-dimensional structures. This method can rapidly screen lead compounds in drug screening and quantitatively analyze the binding affinity between small molecules and target proteins, providing theoretical support for new drug research and development [31,32]. Moreover, molecular dynamics simulation has emerged as a highly appealing technique. It offers crucial perspectives for deciphering the time-dependent movements between macromolecules and small molecules. Currently, this technology is employed to further clarify the binding of pro-drugs to their targets and verify the outcomes of molecular docking. As a result, it enables a more accurate comprehension of the dynamic processes and interactions involved [33,34,35]. Based on these, in this study, the NCI-H1975 cell line was selected as the research target. A comprehensive approach combining cell experiments, molecular docking simulation, and molecular dynamics simulation was adopted to investigate the antitumor activity against NSCLC and elucidate its underlying molecular mechanisms. Our findings aims to provide scientific basis and a potential prodrug to therapy for NSCLC.

## 2. Results

### 2.1. The Antiproliferative Activity of α-Mangostin on NCI-H1975 Cells

**Figure 1:**
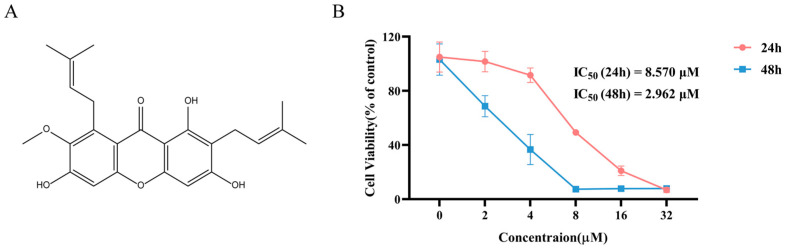
Chemical structure, and cell viability of NCI-H1975 treatment with α-Mangostin. (**A**) The chemical structure of α-Mangostin. (**B**) NCI-H1975 cells were treated with various concentrations of α-Mangostin for 24 h and 48 h. Data represent the average of three independent experiments (means ± SEM).

The chemical structure of α-Mangostin is shown in Figure 1A. To investigate the efficacy of α-Mangostin against NSCLC, an MTT assay was first conducted to assess the inhibitory effect of α-Mangostin on the cell viability of NCI-H1975 cells. As observed in Figure 1B, α-Mangostin inhibited the viability of NCI-H1975 cells in a concentration- and time-dependent manner. The IC_50_ values of α-Mangostin against NCI-H1975 cells were 8.570 µM for 24 h-treatment and 2.962 µM for 48 h-treatment.

### 2.2. The Regulation of α-Mangostin on the EGFR/STAT3 Pathway

**Figure 2:**
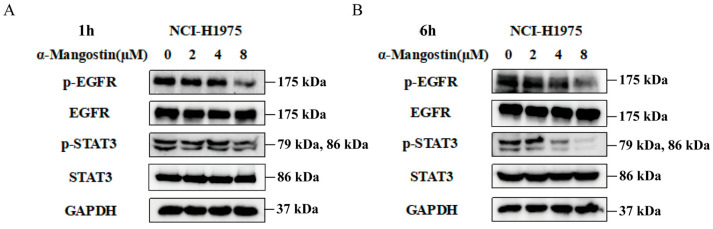
α-Mangostin suppresses phosphorylation of EGFR and STAT3 in a time- and dose- dependent manner. Cells were treated with α-Mangostin (0, 2, 4, 8 µM) for 1 h (**A**) or 6 h (**B**), and then detected by Western blotting.

In order to further investigate whether the antiproliferative effect was mediated by EGFR signaling, we treat NCI-H1975 cells with various doses of α-Mangostin for 1 h or 6 h, and Western blotting analysis was subsequently used to detect the protein expression levels of EGFR and its downstream pathway. As observed in Figure 2A,B, α-Mangostin significantly downregulated the phosphorylation of EGFR and STAT3 in a relatively short time, while the relative total protein expression of the two remained nearly constant. Moreover, the down-regulation was in a time- and dose-dependent manner. Therefore, we conclude that α-Mangostin effectively inhibited the viability of NCI-H1975 cells by directly suppressing the phosphorylation of EGFR and STAT3.

### 2.3. The Proapoptotic Effects of α-Mangostin on NCI-H1975 Cells

**Figure 3:**
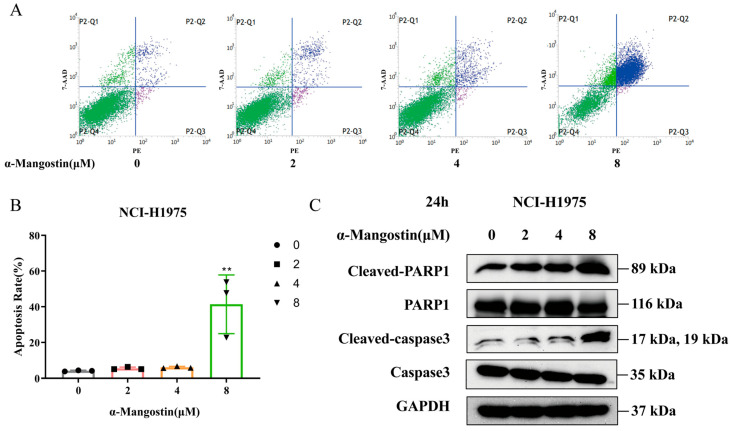
α-Mangostin-induced apoptosis in NCI-H1975 cells. (**A**) Cells were treated with α-Mangostin (0, 2, 4, 8 µM) for 24 h, and then analyzed by using flow cytometry. (**B**) The percentage of apoptotic cells was quantified. (**C**) Expression of PARP, cleaved-PARP1, caspase3 and cleaved-caspase3 was determined by Western blotting. Data represent the average of three independent experiments (means ± SEM). ** *p* < 0.01 versus the control. The data in the figure were analyzed using one-way analysis of variance.

We further assessed the proapoptotic effects of α-Mangostin in NCI-H1975 cells. After 24 h of treatment, Annexin V-PE and 7-AAD double staining were used to detect apoptotic cells. Notably, compared to the control group, the apoptosis rate of NCI-H1975 cells was mostly enhanced with increasing concentrations of α-Mangostin (Figure 3A,B). Meanwhile, we detected the expression of the pro-apoptotic protein by Western blotting. The results showed that the expression levels of cleaved-PARP1 and cleaved-caspase3 were increased in a dose-dependent manner (Figure 3C). These results suggest that α-Mangostin also exerts its antitumor efficacy against NCI-H1975 cells by inducing apoptosis.

### 2.4. Induction of α-Mangostin on Cell Cycle Redistribution of NCI-H1975 Cells

**Figure 4:**
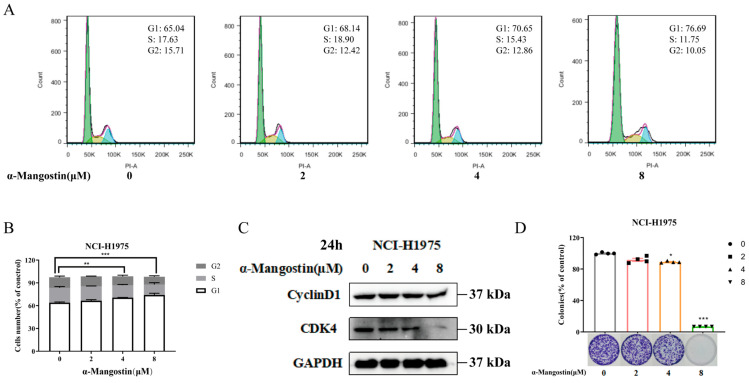
α-Mangostin-induced S-phase cell cycle arrest in NCI-H1975 cells. (**A**) Cells were treated with α-Mangostin (0, 2, 4, 8 µM) for 24 h. Cell cycle distribution was detected by flow cytometry and analyzed using FlowJo software version 7.6. G1, S, and G2 phases were respectively depicted by green, yellow, and blue. (**B**) Quantification of the proportion of cells in each phase of cell cycle. (**C**) Expression of Cyclin D1 and CDK4 was determined using Western blotting. (**D**) Colony formation assay was performed, and the result was quantified. Data represent the average of three independent experiments (means ± SEM). * *p* < 0.05, ** *p* < 0.01, *** *p* < 0.001 versus the control. The data in the figure were analyzed using one-way analysis of variance.

Based on the above-mentioned results, we evaluated whether α-Mangostin could induce cell cycle redistribution in NCI-H1975 cells. We treated NCI-H1975 cells with different concentrations of α-Mangostin for 24 h and then conducted a cell cycle analysis using flow cytometry. The results of Figure 4A,B showed that α-Mangostin treatment resulted in an obvious increase in the number of cells in the G1 phase, accompanied by an increase in treatment concentration. The results of Western blotting indicated that the expression levels of Cyclin D1 and CDK4, which are the key regulators in the G1 expression, were downregulated with an increase in α-Mangostin concentration (Figure 4C). Next, we performed a colony formation assay to further validate the result caused by G1 phase arrest. α-Mangostin was found to markedly restrict colony formation in NCI-H1975 cells with an increase in treatment concentration (Figure 4D). Based on these, we conclude that α-Mangostin causes the G1 phase arrest by regulation of Cyclin D1 and CDK4.

### 2.5. Inhibition of α-Mangostin on the Migration of NCI-H1975 Cells

**Figure 5:**
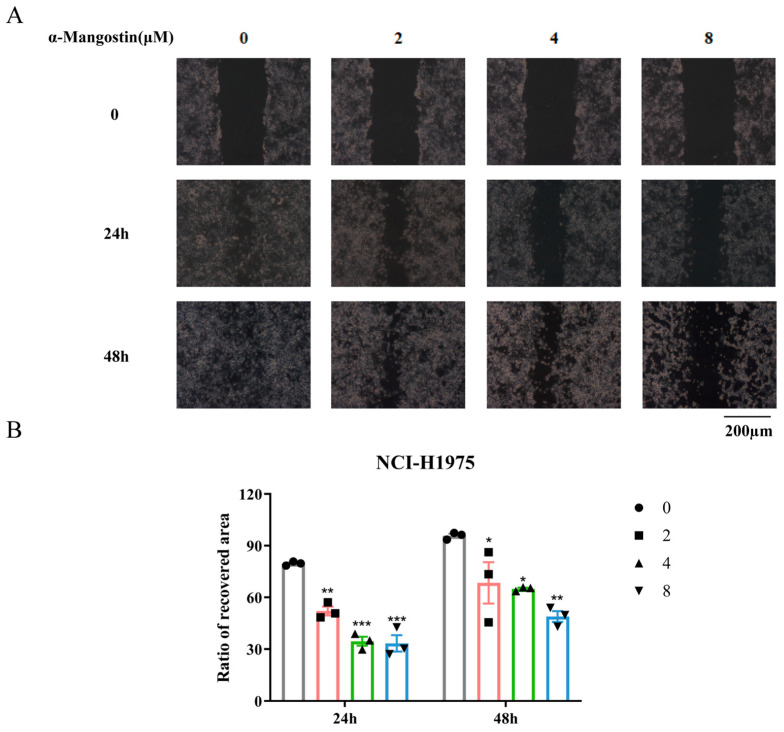
α-Mangostin inhibited cell migration against NCI-H1975 cells. (**A**) Scratches were created and then NCI-H1975 cells were treated with α-Mangostin (0, 2, 4, 8 µM). Multiple images were taken at 0, 24 h and 48 h. (**B**) The quantification of the ratio of the recovered wound area at 24 h and 48 h. Data represent the average of three independent experiments (means ± SEM). * *p* < 0.05, ** *p* < 0.01, *** *p* < 0.001 versus the control. The data in the figure were analyzed using one-way analysis of variance.

A wound healing assay was used to measure the effect of α-Mangostin on the migration of NCI-H1975 cells. In the control group, it could be observed that the NCI-H1975 cells displayed noticeable migration both at 24 h and 48 h, whereas in the α-Mangostin-treated group, the cells appeared to have a delayed healing (Figure 5A,B). These results indicated that α-Mangostin hindered the migration of NCI-H1975 cells to various degrees at different concentrations.

### 2.6. The Binding Mode of α-Mangostin to EGFR and STAT3 in Molecular Docking

**Figure 6:**
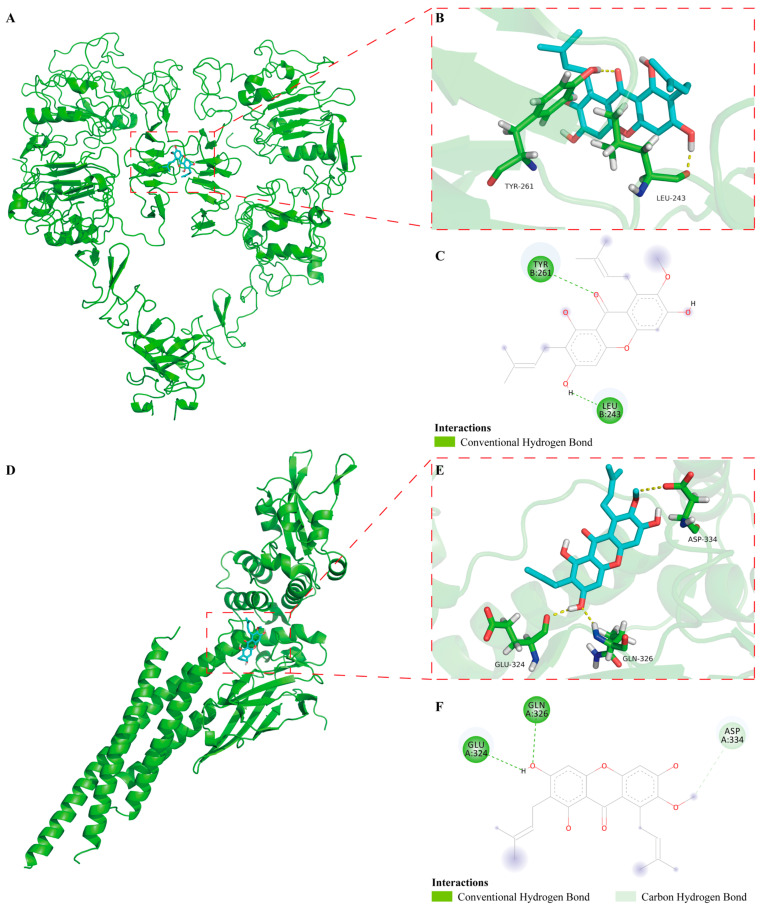
In docking model of α-Mangostin bound to EGFR (PDB code: 3NJP) and STAT3 (PDB code: 6NJS). (**A**) Binding of α-Mangostin to EGFR. EGFR was depicted by green cartoon, while α-Mangostin was shown by blue stick. (**B**) 3D model of α-Mangostin interacted with EGFR. Key residues are shown as sticks and colored by element type (C, green; O, red; N, blue; polar H, white), while hydrogen bonds are shown as yellow dotted lines. (**C**) 2D model of α-Mangostin interacted with EGFR. (**D**) Binding of α-Mangostin to STAT3. (**E**) 3D model of α-Mangostin interacted with STAT3. (**F**) 2D model of α-Mangostin interacted with STAT3.

Molecular docking simulation is usually utilized to effectively predict the binding mode of drugs and molecular targets. Based on it, to further investigate the interaction of α-Mangostin with EGFR and STAT3, we performed molecular docking simulations using AutoDock Tools. The best binding energy for α-Mangostin interacted with EGFR and STAT3 was −4.87 kcal/mol and −4.76 kcal/mol, respectively. The results indicated that the two binding models are credible. As shown in the docking model of α-Mangostin-EGFR (Figure 6A–C), overall, the details of the interaction between α-Mangostin and EGFR, it was observed that α-Mangostin formed two hydrogen bonds with residues of TYR-261 and LEU-243. Meanwhile, the docking model of α-Mangostin-STAT3 showed that α-Mangostin formed three hydrogen bonds with residues of GLU-324, GLN-326 and ASP-334 (Figure 6D–F). The formation of hydrogen bonds helps to firmly anchor the ligand at the specific binding site of the receptor, enhancing the stability of the complex [36]. α-Mangostin forms hydrogen bonds with EGFR at TYR-261 and LEU-243. TYR-261 contains a phenolic hydroxyl group, and its oxygen atom may participate in hydrogen bond formation. This allows α-Mangostin to interact with EGFR continuously, thereby affecting the normal function of EGFR. α-Mangostin forms hydrogen bonds with STAT3 at GLU-324, GLN-326, and ASP-334. Both GLU-324 and ASP-334 contain carboxyl groups, and the oxygen atoms in the carboxyl groups are excellent hydrogen-bond acceptors [37]. This may inhibit STAT3 from entering the nucleus and activating the transcription of downstream target genes. Based on it, the above results indicated that these residues may play critical roles in the regulation of EGFR and STAT3.

### 2.7. The Molecular Dynamics Analysis of the Complexes of α-Mangostin and EGFR or STAT3

**Figure 7:**
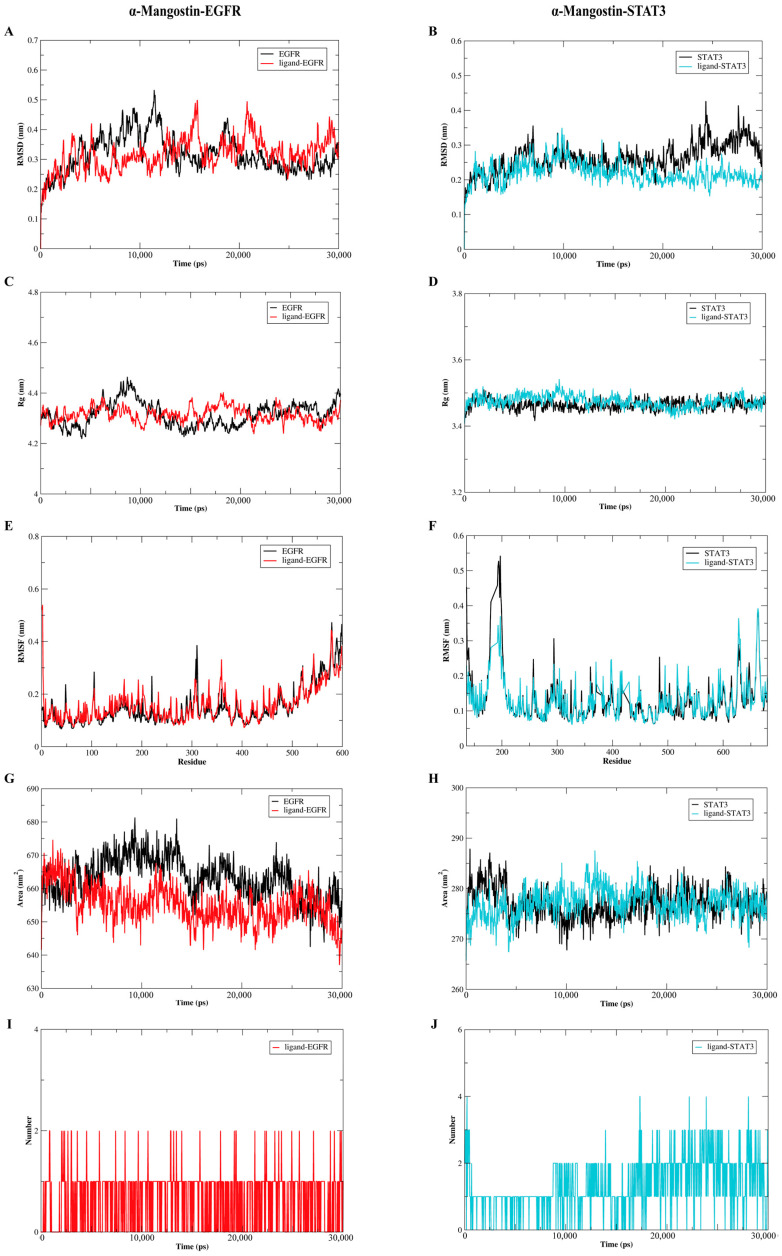
Molecular dynamics simulations were carried out to investigate the interactions between α-Mangostin and either EGFR or STAT3. (**A**) Root-mean-square deviation (RMSD) profiles were presented for free EGFR as well as the α-Mangostin-EGFR complex. (**B**) RMSD profiles were shown for free STAT3 and the α-Mangostin-STAT3 complex. (**C**) Radius of gyration (Rg) profiles were depicted for free EGFR and the α-Mangostin-EGFR complex. (**D**) Rg profiles were illustrated for free STAT3 and the α-Mangostin-STAT3 complex. (**E**) Root-mean-square fluctuation (RMSF) profiles were presented for free EGFR and the α-Mangostin-EGFR complex. (**F**) RMSF profiles were shown for free STAT3 and the α-Mangostin-STAT3 complex. (**G**) Solvent-accessible surface area (SASA) analyses were displayed for free EGFR and the α-Mangostin-EGFR complex. (**H**) SASA analyses were presented for free STAT3 and the α-Mangostin-STAT3 complex. (**I**) The quantity of hydrogen bonds formed between α-Mangostin and EGFR within 30 ns was demonstrated. (**J**) The number of hydrogen bonds between α-Mangostin and STAT3 during 30 ns was exhibited. The experimental data are the average values obtained from three repeated experiments.

To further analyze the binding models of α-Mangostin and EGFR or STAT3, the GROMACS package, which is version 2020.6, was utilized to conduct molecular dynamics simulations spanning 30 ns. As depicted in Figure 7A,B, a comprehensive analysis of the root-mean-square deviation (RMSD) details revealed that in general, the ligand-receptor complexes exhibited less stability fluctuation when compared to the free forms of EGFR or STAT3. The values of the α-Mangostin-EGFR complex, free EGFR, the α-Mangostin-STAT3 complex and free STAT3 were 0.220 ± 0.032 nm, 0.261 ± 0.042 nm, 0.316 ± 0.056 nm, and 0.317 ± 0.062 nm, respectively. As observed in Figure 7C,D, the results of the radius of gyration (Rg) showed that the α-Mangostin-receptor complexes were more stable than those of free EGFR or STAT3 after 20 ns, with the values of 3.475 ± 0.018 nm (α-Mangostin-EGFR complex), 3.465 ± 0.015 nm (EGFR), 4.316 ± 0.029 nm (α-Mangostin-STAT3 complex), and 4.314 ± 0.047 nm (α-Mangostin-STAT3 complex). This suggested that the more compact forms are created after the binding of ligand to proteins. In light of these findings, we carried out an assessment of the root-mean-square fluctuation (RMSF) to evaluate the fluctuations occurring between 20 ns and 30 ns. The results indicated that the α-Mangostin-protein complexes shared a comparable distribution pattern with the corresponding free proteins (as shown in Figure 7E,F). Moreover, the solvent-accessible surface area (SASA) serves as a crucial parameter for reflecting the folding and stability of proteins. The results of Figure 7G,H showed the α-Mangostin-EGFR complex and the α-Mangostin-STAT3 complex form stability with average SASA values of 277.1 ± 2.948 nm^2^ and 655.2 ± 5.631 nm^2^, respectively, as compared with free EGFR or STAT3. Moreover, as presented in Figure 7I,J, the hydrogen bond, which is of great significance for the binding strength of the ligand-receptor complex, progressively strengthens from the initial phase. In the α-Mangostin-EGFR complex, this occurs within the range of 0–2, and in the α-Mangostin-STAT3 complex, it takes place within the range of 0–4. These results reflect that our predicted models are credible.

## 3. Discussion

Among most patients with NSCLC who harbor EGFR-activating mutations, such as deletions in exon 19 (19del) and the L858R mutation, a high initial response rate is observed when treated with first-generation EGFR-TKIs, including gefitinib, erlotinib, and icotinib [38]. Nevertheless, acquired resistance to these inhibitors tends to develop within 9 to 13 months [39]. The most prevalent cause of resistance is the emergence of the secondary EGFR T790M mutation in the kinase domain, which is responsible for more than half of the resistance cases observed [40]. Studies have demonstrated that α-Mangostin plays an important role in resisting various cancers [41,42,43,44]. Furthermore, numerous studies have also reported that α-Mangostin possesses protective efficacy against cytotoxicity induced by cisplatin, which is one of the typical chemotherapeutic drugs for the treatment of lung cancer [24,25,45]. α-Mangostin has been proven to be an effective inhibitor of IDH1 mutations [28]. However, IDH1 mutations are usually absent in NSCLC models. Instead, EGFR mutations are prevalent. Yet, the inhibitory effect of α-Mangostin on EGFR mutations remains unclear. Therefore, we hypothesized that α-Mangostin exerts anti-lung cancer activity and has the advantage of low toxicity to further study its actions and mechanisms against NSCLC harboring EGFR T790M mutation.

EGFR, a prominent target for the treatment of NSCLC, is a transmembrane protein. When EGFR is activated, it triggers the phosphorylation of EGFR and initiates signal transduction in its downstream pathways, such as the RAS/RAF/MEK/ERK, PI3K/AKT, and JAK/STAT pathways [6,46,47]. Among these pathways, the activation of signal transducer and activator of transcription 3 (STAT3) is commonly detected in patients with NSCLC. Moreover, inhibiting STAT3 in NSCLC cell lines significantly undermines their viability [7,40]. Furthermore, it is reported that STAT3 is associated with the acquired resistance to EGFR-TKIs, such as erlotinib [7,48,49]. Therefore, suppression of STAT3 signaling plays a critical role in NSCLC intervention. In this study, we explored the mechanism of α-Mangostin against NCI-H1975 cells. α-Mangostin rapidly suppressed p-EGFR and p-STAT3 at the same time, and the regulation was increased with an increased time. This suggests that α-Mangostin is likely to induce STAT3 signal transduction via EGFR and also directly interact with STAT3. Based on this, we conducted molecular docking and molecular dynamics simulation to the potential interaction between α-Mangostin and EGFR or STAT3. The results indicated that α-Mangostin may bind to EGFR or STAT3 with high binding energies and the predicted models are stable and credible. In the future study, an in vivo assay, validation of STAT3 knockdown, and molecular interaction assays based on surface plasmon resonance (SPR) or bio-layer interferometry (BLI) technology need to be performed to further validate these findings.

Apoptosis, a form of programmed cell death, is regulated by signaling pathways initiated by diverse stimulating factors, including cell stress, DNA damage, and immune surveillance [50,51]. Apoptosis induction stands as a key strategy in cancer therapy. In this present research, we discovered that α-Mangostin triggered apoptosis and suppressed the proliferation of NCI-H1975 cells. Apoptosis induction serves as a crucial mechanism contributing to the therapeutic effectiveness of epidermal growth factor receptor (EGFR)-tyrosine kinase inhibitors (TKIs) in non-small cell lung cancer (NSCLC) cases with EGFR mutations. In this study, we showed that α-Mangostin facilitates apoptosis in NSCLC cells carrying the EGFR T790M mutation. Additionally, we observed that α-Mangostin led to cell cycle arrest by modulating CDK4 and cyclin D1. To further validate this finding, a colony formation assay was conducted. Since cell cycle arrest inhibits clonogenic formation, this assay was used to assess the impact of the treatment on the ability of cells to form colonies. The results of the colony formation assay were consistent with the results of the cell cycle assay. In addition, α-Mangostin also delayed the migration of NCI-H1975 cells. In molecular docking, α-Mangostin can bind to the active pockets of both EGFR and STAT3. The results of molecular dynamics also demonstrate the stability of this complex. Based on these results, we deduced that α-Mangostin acts on NCI-H1975 cells and may be an effective strategy for the treatment of NSCLC. Based on the results of this experiment, in our future research, we plan to conduct experiments on multiple cell lines with different activating mutations of EGFR. Our aim is to explore the universality of α-Mangostin in treating NSCLC by acting on EGFR mutations.

## 4. Materials and Methods

### 4.1. Materials

α-Mangostin was purchased from Chengdu Biopurify Phytochemicals Ltd. (Chengdu, China). As stated in the certificate of analysis, the purity of α-Mangostin exceeded 98.7%. The NCI-H1975 cell line was obtained from Cell Bank of Kunming (Kunming, China). Dimethylthiazol-2-yl)-2,5-diphenyltetrazolium bromide (MTT) was purchased from Sigma-Aldrich (St. Louis, MO, USA). All primary antibodies were purchased from Cell Signaling Technology (Danvers, MA, USA). All secondary antibodies were purchased from Thermo Fisher Scientific (Waltham, MA, USA).

### 4.2. Cell Culture

The NCI-H1975 cells were cultured in RPMI 1640 medium (MeilunBio, Dalian, China) supplemented with 10% fetal bovine serum (Viva cells), 10 U/mL of penicillin and 0.1 mg/mL streptomycin (MeilunBio, Dalian, China). The cells were maintained in a humidified atmosphere containing 5% CO_2_ at 37 °C. The culture medium was replaced for approximately 1–2 days and the cells were detached using 0.25% trypsin-EDTA (no phenol red) (MeilunBio, Dalian, China).

### 4.3. MTT Assay

NCI-H1975 cells were seeded in a 96-well plate at a density of 2 × 10^4^ cells/well and incubated overnight at 37 °C. The cells were then washed twice with PBS (Solarbio, Beijing, China) before removing the culture medium and replacing it with 200 µL serum-free medium diluted with multiple concentrations (0, 2, 4, 8, 16, 32 µM) of α-Mangostin. After 24 or 48 h, the medium was renewed after washing the cells with PBS, followed by the addition of 20 µL MTT to each well and incubated for 4 h. The medium was gently removed, and 150 µL Dimethylsulfoxide (DMSO) was added to each well, and absorbance was measured at 492 nm on a microplate reader. The IC_50_ values were calculated using GraphPad Prism 5.0.

### 4.4. Western Blotting

The protein was extracted using a 100:1 mixture of RIPA buffer and PMSF (Solarbio, Beijing, China). After obtaining the total protein, the samples were processed by SDS-PAGE, and then the proteins were electrotransferred onto PVDF membranes (EMD Millipore, Bedford, MA, USA). To prevent non-specific binding, the membranes were blocked in TBS containing 5% non-fat milk and 0.1% Tween-20 at room temperature for 1 h and then incubated overnight at 4 °C with the following primary antibodies. Different antibodies use different membranes respectively. Thereafter, the membranes were incubated with secondary antibodies at RT for 1 h and the immunoblots were visualized using FluorChem E System (ProteinSimple, San Jose, CA, USA).

### 4.5. Cell Apoptosis Assay

An Annexin V-PE/7-AAD apoptosis detection kit from MeilunBio (Dalian, China) was utilized to investigate the pro-apoptotic effect. Initially, 1 × 10^6^ cells were seeded onto 60-mm culture plates and incubated overnight. Subsequently, the culture medium was replaced with serum-free medium containing various concentrations of α-Mangostin (0, 2, 4, 8 µM). Following a 24 h treatment, the cells were resuspended in 100 µL of 1× binding buffer. Then, 5 µL of Annexin-PE and 10 µL of 7-AAD were added to each tube and incubated in the dark at room temperature for 15 min for staining. After staining, 400 µL of 1× binding buffer was added, the mixture was thoroughly mixed, and the samples were analyzed using a flow cytometer (BD FACSJazz TM Cell Sorter, BD Biosciences, San Jose, CA, USA).

### 4.6. Cell Cycle Assay

The assessment of cell cycle arrest was carried out using a cell cycle detection kit from 4A Biotech (Beijing, China). The cells were seeded on 60-mm culture dishes at a density of 2 × 10^6^ cells per dish. They were then exposed to either the control medium or medium supplemented with different concentrations (2, 4, 8 µM) of α-Mangostin for 24 h. After the treatment, the cells were fixed in 75% chilled ethanol at −20 °C for 24 h. Subsequently, the fixed cells were washed twice with PBS. For staining, a mixture was prepared for each sample, consisting of 400 µL of binding buffer, 15 µL of PI (25×), and 4 µL of RNase A (2.5 mg/mL). The cells were incubated with this staining solution at 37 °C in the dark for 30 min. Flow cytometry (BD FACSCalibur, BD Biosciences, San Jose, CA, USA) was employed to analyze the cell cycle distribution.

### 4.7. Colony Formation Assay

NCI-H1975 cells were seeded on 60-mm culture dishes at 1000 cells per dish and treated with indicated α-Mangostin concentrations (0, 2, 4, 8 µM) for 72 h. The culture medium changed every three days, the process lasting approximately 11 days. The cells were fixed with paraformaldehyde and stained with 0.01% crystal violet solution. Images were taken, clones were dissolved in 10% glacial acetic acid, and absorbance was measured at 560 nm using a microplate reader.

### 4.8. Wound Healing Assay

NCI-H1975 cells were seeded at 2 × 10^6^ cells per 60-mm culture dish. A scratch wound was made by scraping the dish with a 200-µL pipette tip. The cells were washed three times to remove floating debris. The cells were treated with α-Mangostin at concentrations 0, 2, 4, 8 µM. Images at 0 and 24 h acquired using an Olympus CKX41 biomicroscope. The image data were analyzed with ImageJ software version 1.52.

### 4.9. Molecular Docking

The crystal structures of EGFR (PDB code: 3NJP) and STAT3 (PDB code: 6NJS) were retrieved from the Protein Data Bank (http://www.rcsb.org, accessed on 1 March 2024) and served as receptors for docking. The DiscoveryStudio visualizer (version 2016) was employed to pre-process both ligands and receptors. The structure of α-Mangostin was acquired from PubChem (https://pubchem.ncbi.nlm.nih.gov/, accessed on 1 March 2024) to act as a small-molecule ligand. As per the literature, AutoDock Tools (version 1.56) was utilized for grid generation and docking operations. The docking parameters were mostly set to their default values. However, the number of genetic algorithm (GA) runs was set to 20, and the maximum number of events (medium) was set at 5,000,000. Ultimately, the docking pose with the lowest binding energy was chosen. Subsequently, the interaction between the receptor and the ligand was further visualized using the DiscoveryStudio visualizer and PyMol software (version 1.5).

### 4.10. Molecular Dynamics Simulation

The optimal models of α-Mangostin-EGFR and α-Mangostin-STAT3 obtained from the results of molecular docking for the complexes of α-Mangostin combined with receptors were utilized to perform molecular dynamics simulations using the GROMACS-2020.6 package. The force field named “AMBERSB99” and the simple point charge (SPC) water model was selected to assign the ligand and EGFR or STAT3, respectively. Subsequently, the surface charges for the complexes of α-Mangostin-EGFR and α-Mangostin-STAT3 were configured by adding counterions (Na^+^ or Cl^−^). Sixteen sodium ions (Na^+^) were added to both the α-Mangostin-epidermal growth factor receptor (EGFR) complex and free EGFR. Four chloride ions (Cl^−^) were added to both the α-Mangostin-signal transducer and activator of transcription 3 (STAT3) complex and free STAT3. After that, energy minimization was carried out to relax all atoms. The steepest-descent method and the conjugate-gradient method were employed, with 5000 cycles for each method respectively. Next, the minimized system was slowly heated in a normal volume and temperature (NVT) ensemble. The temperature was raised from 0 to 300 K over a period of 1 ns. Then, it was subjected to a density-equilibration ensemble under normal pressure and temperature (NPT) conditions at 300 K for 1 ns. Ultimately, the molecular dynamics (MD) system was initiated and run for 30 ns with a time step of 2.0 fs.

### 4.11. Statistical Analysis

Data are presented as mean ± standard error of the mean (SEM). All experiments were performed independently at least thrice. Statistical significance was set at *p* < 0.05.

## 5. Conclusions

In summary, our findings demonstrated that α-Mangostin exerts its antitumor effect, including antiproliferation, inducing cell cycle arrest and a pro-apoptosis effect and inhibiting migration against NCI-H1975 cells. These effects are associated with the stable binding of α-Mangostin to EGFR and STAT3 through the formation of hydrogen bonds, leading to the suppression of EGFR/STAT3 signaling. Our results will hopefully provide a prospective therapeutic candidate for patients with NSCLC harboring the EGFR T790M mutation.

## Data Availability

Data will be made available on request.

## References

[1] Siegel R.L., Miller K.D., Wagle N.S., Jemal A. (2023). Cancer Statistics, 2023. CA Cancer J. Clin..

[2] Siegel R.L., Miller K.D., Fuchs H.E., Jemal A. (2022). Cancer statistics, 2022. CA Cancer J. Clin..

[3] Thai A.A., Solomon B.J., Sequist L.V., Gainor J.F., Heist R.S. (2021). Lung cancer. Lancet.

[4] Sabbah D.A., Hajjo R., Sweidan K. (2020). Review on Epidermal Growth Factor Receptor (EGFR) Structure, Signaling Pathways, Interactions, and Recent Updates of EGFR Inhibitors. Curr. Top. Med. Chem..

[5] Harrison P.T., Vyse S., Huang P.H. (2020). Rare epidermal growth factor receptor (EGFR) mutations in non-small cell lung cancer. Semin. Cancer Biol..

[6] Sun P.Y., Zhang S.G., Qu Y.N., Li X.Y., Chen G.R., Wang X.J., Sheng J., Wang J. (2025). Coccinic acid exhibits anti-tumor efficacy against NSCLC harboring EGFR L858R/T790M mutation via the EGFR/STAT3 pathway. Bioorg. Chem..

[7] Yin Z.J., Jin F.G., Liu T.G., Fu E.Q., Xie Y.H., Sun R.L. (2011). Overexpression of STAT3 potentiates growth, survival, and radioresistance of non-small-cell lung cancer (NSCLC) cells. J. Surg. Res..

[8] Sun X.L., Xiang Z.M., Xie Y.R., Zhang N., Wang L.X., Wu Y.L., Zhang D.Y., Wang X.J., Sheng J., Zi C.T. (2022). Dimeric-(-)-epigallocatechin-3-gallate inhibits the proliferation of lung cancer cells by inhibiting the EGFR signaling pathway. Chem. Biol. Interact..

[9] Baumgartner U., Berger F., Hashemi Gheinani A., Burgener S.S., Monastyrskaya K., Vassella E. (2018). miR-19b enhances proliferation and apoptosis resistance via the EGFR signaling pathway by targeting PP2A and BIM in non-small cell lung cancer. Mol. Cancer..

[10] Chen S.F., Zhang Z.Y., Zhang J.L. (2017). Matrine increases the inhibitory effects of afatinib on H1975 cells via the IL-6/JAK1/STAT3 signaling pathway. Mol. Med. Rep..

[11] Mao D., Feng L., Gong H. (2018). The Antitumor and Immunomodulatory Effect of Yanghe Decoction in Breast Cancer Is Related to the Modulation of the JAK/STAT Signaling Pathway. Evid. Based Complement. Altern. Med..

[12] Yang L., Zhou F., Zhuang Y., Liu Y., Xu L., Zhao H., Xiang Y., Dai X., Liu Z., Huang X. (2021). Acetyl-bufalin shows potent efficacy against non-small-cell lung cancer by targeting the CDK9/STAT3 signalling pathway. Br. J. Cancer.

[13] Yu H., Lee H., Herrmann A., Buettner R., Jove R. (2014). Revisiting STAT3 signalling in cancer: New and unexpected biological functions. Nat. Rev. Cancer.

[14] Sun P., Qu Y., Wang Y., Wang J., Wang X., Sheng J. (2021). Wighteone exhibits an antitumor effect against EGFR L858R/T790M mutation non-small cell lung cancer. J. Cancer.

[15] Chen G., Li Y., Wang W., Deng L. (2018). Bioactivity and pharmacological properties of α-Mangostin from the mangosteen fruit: A review. Expert Opin. Ther. Pat..

[16] Zhang K.J., Gu Q.L., Yang K., Ming X.J., Wang J.X. (2017). Anticarcinogenic Effects of α-Mangostin: A Review. Planta Med..

[17] Yang A., Liu C., Wu J., Kou X., Shen R. (2021). A review on α-Mangostin as a potential multi-target-directed ligand for Alzheimers disease. Eur. J. Pharmacol..

[18] Wang F., Ma H., Liu Z., Huang W., Xu X., Zhang X. (2017). α-Mangostin inhibits DMBA/TPA-induced skin cancer through inhibiting inflammation and promoting autophagy and apoptosis by regulating PI3K/Akt/mTOR signaling pathway in mice. Biomed. Pharmacother..

[19] Lee C.H., Ying T.H., Chiou H.L., Hsieh S.C., Wen S.H., Chou R.H., Hsieh Y.H. (2017). Alpha-Mangostin induces apoptosis through activation of reactive oxygen species and ASK1/p38 signaling pathway in cervical cancer cells. Oncotarget.

[20] Zhu X., Li J., Ning H., Yuan Z., Zhong Y., Wu S., Zeng J.Z. (2021). α-Mangostin Induces Apoptosis and Inhibits Metastasis of Breast Cancer Cells via Regulating RXRα-AKT Signaling Pathway. Front. Pharmacol..

[21] Ittiudomrak T., Puthong S., Roytrakul S., Chanchao C. (2019). α-Mangostin and Apigenin Induced Cell Cycle Arrest and Programmed Cell Death in SKOV-3 Ovarian Cancer Cells. Toxicol. Res..

[22] Yang S., Zhou F., Dong Y., Ren F. (2021). α-Mangostin Induces Apoptosis in Human Osteosarcoma Cells Through ROS-Mediated Endoplasmic Reticulum Stress via the WNT Pathway. Cell Transplant..

[23] Phan T., Shahbazzadeh F., Kihara T. (2020). Alpha-Mangostin reduces mechanical stiffness of various cells. Hum. Cell.

[24] Li Q., Yan X.T., Zhao L.C., Ren S., He Y.F., Liu W.C., Wang Z., Li X.D., Jiang S., Li W. (2020). α-Mangostin, a Dietary Xanthone, Exerts Protective Effects on Cisplatin-Induced Renal Injury via PI3K/Akt and JNK Signaling Pathways in HEK293 Cells. ACS Omega.

[25] Pérez-Rojas J.M., Cruz C., García-López P., Sánchez-González D.J., Martínez-Martínez C.M., Ceballos G., Espinosa M., Meléndez-Zajgla J., Pedraza-Chaverri J. (2009). Renoprotection by alpha-Mangostin is related to the attenuation in renal oxidative/nitrosative stress induced by cisplatin nephrotoxicity. Free Radic. Res..

[26] Fei X., Jo M., Lee B., Han S.B., Lee K., Jung J.K., Seo S.Y., Kwak Y.S. (2014). Synthesis of xanthone derivatives based on α-Mangostin and their biological evaluation for anti-cancer agents. Bioorg. Med. Chem. Lett..

[27] Jung H.A., Su B.N., Keller W.J., Mehta R.G., Kinghorn D. (1970). Antioxidant xanthones from the pericarp of Garcinia mangostana (Mangosteen). J. Agric. Food. Chem..

[28] Kim H.J., Fei X., Cho S.C., Choi B.Y., Ahn H.C., Lee K., Seo S.Y., Keum Y.S. (2015). Discovery of α-Mangostin as a novel competitive inhibitor against mutant isocitrate dehydrogenase-1. Bioorg. Med. Chem. Lett..

[29] Phan T., Shahbazzadeh F., Pham T., Kihara T. (2018). Alpha-Mangostin inhibits the migration and invasion of A549 lung cancer cells. PeerJ..

[30] Wang X., Wang Z.Y., Zheng J.H., Li S. (2021). TCM network pharmacology: A new trend towards combining computational, experimental and clinical approaches. Chin. J. Nat. Med..

[31] Aydın S.G. (2022). Review on Molecular Modeling and Docking. SAR J. Sci. Res..

[32] Dudhat K. (2023). Panoramic Review on Progress and Development of Molecular Docking. Pharm. Sci. Technol..

[33] Tuo Y., Lu X., Tao F., Tukhvatshin M., Xiang F., Wang X., Shi Y., Lin J., Hu Y. (2024). The Potential Mechanisms of Catechins in Tea for Anti-Hypertension: An Integration of Network Pharmacology, Molecular Docking, and Molecular Dynamics Simulation. Foods.

[34] Hildebrand P.W., Rose A.S., Tiemann J.K.S. (2019). Bringing Molecular Dynamics Simulation Data into View. Trends Biochem. Sci..

[35] Zhang S.G., Yang Y.F., Zhang R.H., Gao J., Wu M.Y., Wang J., Sheng J., Sun P.Y. (2024). The Potential Mechanism of Alpiniae oxyphyllae Fructus Against Hyperuricemia: An Integration of Network Pharmacology, Molecular Docking, Molecular Dynamics Simulation, and In Vitro Experiments. Nutrients.

[36] Zazeri G., Povinelli A.P.R., Pavan N.M., Jones A.M., Ximenes V.F. (2023). Solvent-Induced Lag Phase during the Formation of Lysozyme Amyloid Fibrils Triggered by Sodium Dodecyl Sulfate: Biophysical Experimental and In Silico Study of Solvent Effects. Molecules.

[37] Zazeri G., Povinelli A.P.R., Le D.C.S., Tang B., Cornelio M.L., Jones A.M. (2020). Synthesis and Spectroscopic Analysis of Piperine- and Piperlongumine-Inspired Natural Product Scaffolds and Their Molecular Docking with IL-1β and NF-κB Proteins. Molecules.

[38] Herbst R.S., Morgensztern D., Boshoff C. (2018). The biology and management of non-small cell lung cancer. Nature.

[39] Zappa C., Mousa S.A. (2016). Non-small cell lung cancer: Current treatment and future advances. Transl. Lung Cancer Res..

[40] Pao W., Miller V.A., Politi K.A., Riely G.J., Somwar R., Zakowski M.F., Kris M.G., Varmus H. (2005). Acquired resistance of lung adenocarcinomas to gefitinib or erlotinib is associated with a second mutation in the EGFR kinase domain. PLoS Med..

[41] Tangsuksan P., Chuerduangphui J., Takahashi Y.C., Srichana T., Hitakomate E., Pientong C., Ekalaksananan T., Nittayananta W. (2021). Mucoadhesive film containing α-Mangostin shows potential role in oral cancer treatment. BMC Oral Health.

[42] Li R.R., Zeng D.Y. (2021). The effects and mechanism of α-Mangostin on chemosensitivity of gastric cancer cells. Kaohsiung J. Med. Sci..

[43] Chien H.J., Ying T.H., Hsieh S.C., Lin C.L., Yu Y.L., Kao S.H., Hsieh Y.H. (2020). α-Mangostin attenuates stemness and enhances cisplatin-induced cell death in cervical cancer stem-like cells through induction of mitochondrial-mediated apoptosis. J. Cell. Physiol..

[44] Herdiana Y., Wathoni N., Shamsuddin S., Muchtaridi M. (2021). α-Mangostin Nanoparticles Cytotoxicity and Cell Death Modalities in Breast Cancer Cell Lines. Molecules.

[45] Reyes-Fermín L.M., Avila-Rojas S.H., Aparicio-Trejo O.E., Tapia E., Rivero I., Pedraza-Chaverri J. (2019). The Protective Effect of Alpha-Mangostin against Cisplatin-Induced Cell Death in LLC-PK1 Cells is Associated to Mitochondrial Function Preservation. Antioxidants.

[46] Marmor M.D., Skaria K.B., Yarden Y. (2004). Signal transduction and oncogenesis by ErbB/HER receptors. Int. J. Radiat. Oncol. Biol. Phys..

[47] Reungwetwattana T., Weroha S.J., Molina J.R. (2012). Oncogenic pathways, molecularly targeted therapies, and highlighted clinical trials in non-small-cell lung cancer (NSCLC). Clin. Lung Cancer..

[48] Tang Y., Wang Y., Wang X., Zhao Z., Cai H., Xie M., Jiang X., Zhang L., Cheng J., Yang L. (2022). Acetylshikonin exerts anti-tumor effects on non-small cell lung cancer through dual inhibition of STAT3 and EGFR. Phytomedicine.

[49] Shen Y., Cai H., Ma S., Zhu W., Zhao H., Li J., Ye H., Yang L., Zhao C., Huang X. (2022). Telocinobufagin Has Antitumor Effects in Non-Small-Cell Lung Cancer by Inhibiting STAT3 Signaling. J. Nat. Prod..

[50] Carneiro B.A., El-Deiry W.S. (2020). Targeting apoptosis in cancer therapy. Nat. Rev. Clin. Oncol..

[51] Pistritto G., Trisciuoglio D., Ceci C., Garufi A., D’Orazi G. (2016). Apoptosis as anticancer mechanism: Function and dysfunction of its modulators and targeted therapeutic strategies. Aging.

